# Congenital Disorders of Glycosylation: What Clinicians Need to Know?

**DOI:** 10.3389/fped.2021.715151

**Published:** 2021-09-03

**Authors:** Patryk Lipiński, Anna Tylki-Szymańska

**Affiliations:** Department of Pediatrics, Nutrition and Metabolic Diseases, The Children's Memorial Health Institute, Warsaw, Poland

**Keywords:** congenital disorders of glycosylation, clinical presentation, isoelectric focusing of serum transferrin, next-generation sequencing, treatment

## Abstract

Congenital disorders of glycosylation (CDG) are a group of clinically heterogeneous disorders characterized by defects in the synthesis of glycans and their attachment to proteins and lipids. This manuscript aims to provide a classification of the clinical presentation, diagnostic methods, and treatment of CDG based on the literature review and our own experience (referral center in Poland). A diagnostic algorithm for CDG was also proposed. Isoelectric focusing (IEF) of serum transferrin (Tf) is still the method of choice for diagnosing N-glycosylation disorders associated with sialic acid deficiency. Nowadays, high-performance liquid chromatography, capillary zone electrophoresis, and mass spectrometry techniques are used, although they are not routinely available. Since next-generation sequencing became more widely available, an improvement in diagnostics has been observed, with more patients and novel CDG subtypes being reported. Early and accurate diagnosis of CDG is crucial for timely implementation of appropriate therapies and improving clinical outcomes. However, causative treatment is available only for few CDG types.

## Background

Congenital disorders of glycosylation (CDG), previously known as carbohydrate-deficient glycoprotein syndromes, constitute a group of inborn errors of metabolism (IEM) characterized by impaired synthesis and attachment of glycans to glycoproteins and glycolipids and impaired synthesis of glycosylphosphatidylinositol. Two main types of protein glycosylation are depicted, including N-glycosylation and O-glycosylation, while N-glycosylation is the most common type in the human body. So far, more than 150 CDG subtypes have been reported, while the phosphomannomutase-2 deficiency (PMM2-CDG) comprises the most common one (1–4).

Currently, CDG types are classified into defects in protein N-glycosylation, protein O-glycosylation, glycosphingolipid, and glycosylphosphatidylinositol (GPI) anchor glycosylation defects, and multiple glycosylation pathway defects (1–4).

CDG nomenclature is denoted by the affected gene name (non-italicized) followed by -CDG (i.e., PMM2-CDG, phosphomannomutase-2 deficiency) (1–4).

Most CDG types are autosomal recessive in inheritance, but autosomal dominant (i.e., EXT1/EXT2-CDG, GANAB-CDG, PRKCSH-CDG, POGLUT1-CDG, POFUT1-CDG) as well as X-linked (i.e., ALG13-CDG, PIGA-CDG, SLC35A2-CDG, ATP6AP1-CDG) forms have also been described (1–4).

This manuscript aims to provide a classification of the clinical presentation, diagnostic methods, and treatment of CDG based on the literature review and our own experience (referral center in Poland). A diagnostic algorithm for CDG was also proposed.

## Clinical Presentation

CDG are usually multisystem diseases with neurological manifestation observed in most patients (5, 6). Like in other IEM, depending on the disease severity (age of symptom onset), mild to severe phenotypes could be observed.

Most CDG patients presenting with an early-onset neurovisceral phenotype have some signs and symptoms since birth. Thus, a detailed clinical analysis, including a physical examination (i.e., craniofacial dysmorphia, birth body length, weight, and head circumference) as well as family and pregnancy history (i.e., regarding non-immune hydrops fetalis, NIHF), is essential in the context of further biochemical and molecular analyses. NIHF was commonly reported in PMM2-CDG, ALG9-CDG, and ALG8-CDG, and the presence of NIHF is associated with poor outcomes (7).

Neurological signs and symptoms include psychomotor retardation, hypotonia, microcephaly, epileptic seizures, ataxia, peripheral neuropathy, and stroke-like episodes (5, 6). Deficiencies in several glycosylation pathways comprise the cause of epilepsy, while in some of them (i.e., ALG1-CDG, ALG3-CDG, ALG11-CDG, ALG13-CDG, DPM1-CDG, DPM2-CDG, MPDU1-CDG, DPAGT1-CDG, RFT1-CDG, PIGA-CDG, PIGW-CDG, and PIGQ-CDG) the severe epileptic encephalopathies have been described (8–23). Besides cerebellar and cerebral atrophy, most CDG patients with epilepsy do not have characteristic brain malformations. However, O-glycosylation disorders are associated with neuronal migration defects, including lissencephaly, polymicrogyria, schizencephaly, and neuronal heterotopia (24). The cerebellum is commonly affected in PMM2-CDG, dystroglycanopathies, and SRD5A3-CDG, while the course of cerebellar ataxia is not progressive (25–28). Several CDG types, especially dystroglycanopathies, are connected with congenital muscular dystrophy (29–33).

In the majority of CDG, liver involvement is observed as a part of multisystem phenotype, presenting with elevated serum transaminases (more often) and hepatomegaly (less often) in early infancy/childhood, while serum transaminases could normalize later in life (34–36). In the case of severe neurovisceral phenotype leading to premature death, severe liver involvement is observed as part of multiple organ failure (i.e., COG7-CDG, ALG3-CDG) (37). There is also a group of CDG, including MPI-CDG, CCDC115-CDG, and TMEM199-CDG, in which the disease is expressed mainly in the liver (no neurological manifestation) (38–43). There is no typical histologic pattern for liver disease in CDG; liver fibrosis, or even cirrhosis, was reported in PMM2-CDG, MPI-CDG, and TMEM199-CDG (44).

About 20% of CDG were reported to exhibit heart disease in the form of pericardial effusion, cardiomyopathy, arrhythmias, and structural abnormalities (45, 46). Structural (valvular and septal) defects are predominant in patients with GPI-anchor biosynthesis defects and COG-CDG (47–49). Pericardial effusions are characteristic features of PMM2-CDG, while dilated cardiomyopathy is typical for PGM1-CDG and DK1-CDG(50–52).

Recurrent and severe infections as a part of immunodeficiency phenotype were reported in ALG12-CDG, ATP6AP1-CDG, EXTL3-CDG, G6PC3-CDG, MOGS-CDG, PGM3-CDG, and SLC35C1-CDG (53–60).

Some types of CDG, including ALG3-CDG, ALG6-CDG, ALG9-CDG, ALG12-CDG, PGM3-CDG, CSGALNACT1-CDG, SLC35D1-CDG, and TMEM-165, were reported with well-defined skeletal dysplasia (61–69). In addition, some skeletal abnormalities are also unique for some types of CDG, including Schneckenbecken dysplasia in SLC35D1-CDG, brachytelephalangy in PIGV-CDG and PIGO-CDG, pseudodiastrophic dysplasia in ALG12-CDG, Gillessen-Kaesbach and Nishimura skeletal dysplasia in ALG9-CDG, and Desbuquois dysplasia in PGM3-CDG (48, 70–72).

Some CDG also have unique characteristics in the form of a constellation of clinical symptoms), which may facilitate their recognition and shorten the diagnostic process, including:

- connective tissue involvement (*cutis laxa* in ATP6V0A2-CDG, COG7-CDG; inguinal hernias in ATP6AP1-CDG) (73–76);
- midline malformations, including palate/uvula cleft in PGM1-CDG; a constellation of congenital malformations, dilated cardiomyopathy, liver involvement, variable endocrine, and hematological abnormalities and no neurological disease in PGM1-CDG (38, 77);
- inverted nipples and abnormal fat distribution in PMM2-CDG; specific craniofacial dysmorphia in PMM2-CDG, including microcephaly, prominent forehead, flat nasal bridge, thin upper lip, and large ears; polycystic kidney disease and hyperinsulinemic hypoglycemia in PMM2-CDG due to a promotor defect (26, 78–80);
- cerebellar hypoplasia in PMM2-CDG, SRD5A3-CDG, dystroglycanopathies (25, 28);
- cerebellar ataxia and variable eye malformations, including optic disc hypoplasia and nystagmus in SRD5A3-CDG (28);
- achalasia and alacrima without adrenal insufficiency in GMPPA-CDG (81);
- severe immunodeficiency accompanied by a skeletal dysplasia in PGM3-CDG; immunodeficiency with the Bombay blood phenotype and severe growth and psychomotor retardation in leukocyte adhesion deficiency type II (known as SLC35C1-CDG) (57–59, 82, 83).

## Diagnostic Process

Since the introduction by Jaeken et al. (84), isoelectric focusing (IEF) of serum transferrin (Tf) is the method of choice for diagnosis of hypo-*N*-glycosylation disorders associated with sialic acid deficiency (1–4, 84, 85).

So far, several other laboratory techniques have been used for the separation and quantification of serum Tf isoforms, including high-performance liquid chromatography (HPLC), capillary zone electrophoresis (CZE), and mass spectrometry (MS) (86–94). Every diagnostic method has its own limitations. Both CZE and HPLC techniques are universal with low maintenance cost and suitable for CDG screening. An abnormal result should be further investigated by serum Tf IEF. In addition, serum Tf IEF is the most commonly used for diagnosis and monitoring of CDG, and thus considered as the reference method. Other techniques, including CZE and HPLC, can be adapted by the laboratories based on their equipment accessibility.

IEF of serum Tf from dried blood spot (DBS) samples was recently demonstrated as a reliable method for CDG screening (95, 96). DBS is firmly established in the analysis of various IEM, especially in the context of newborn screening programs across the world. However, there is a number of CDG for which there is no data regarding glycosylation abnormalities after birth and thus further studies are needed.

**Figure 1:**
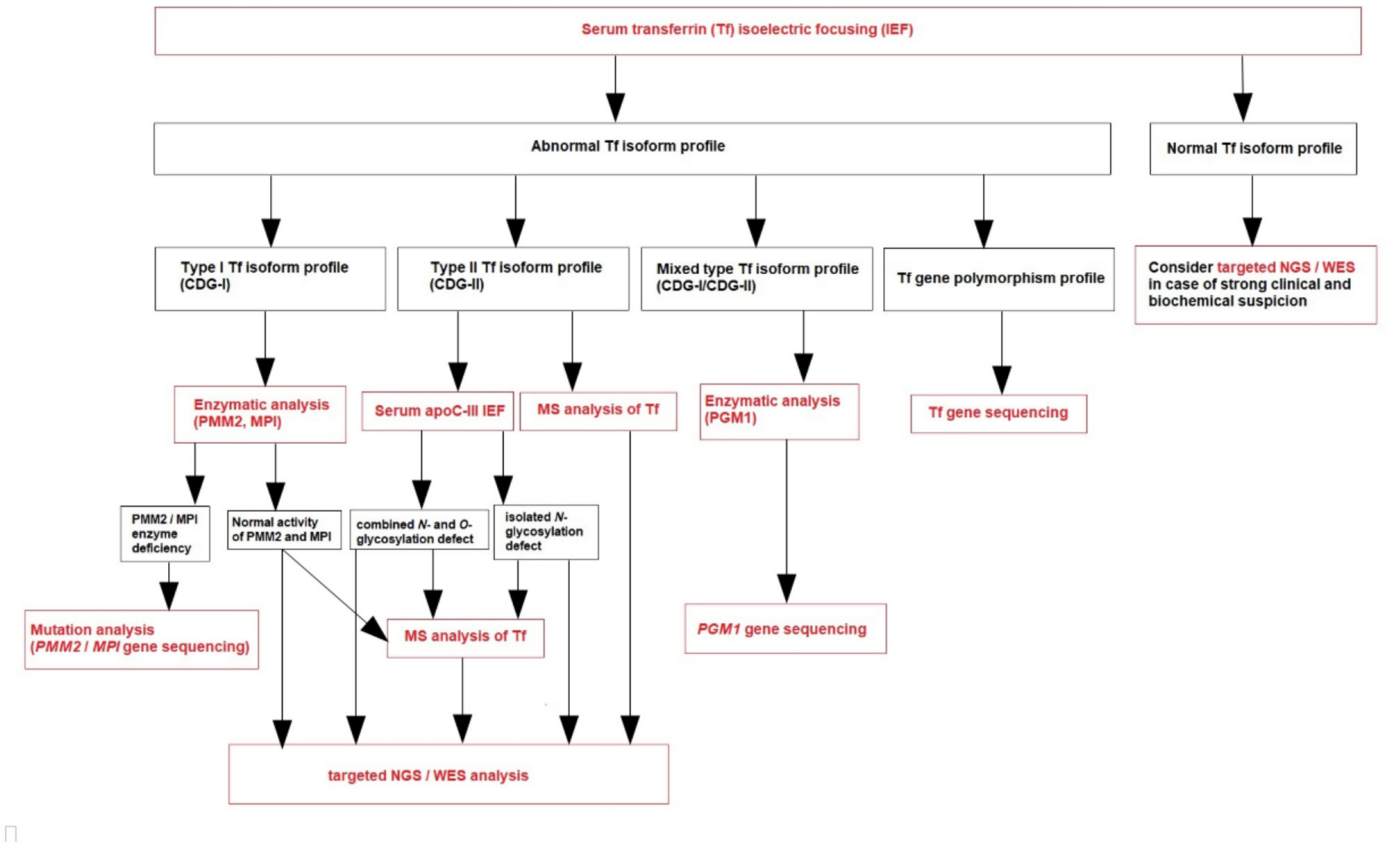
Diagnostic algorithm for CDG.

Based on our own experience (referral center in Poland), we create a diagnostic algorithm for CDG (Figure 1). We recommend to perform serum Tf IEF for an initial screening of glycosylation abnormalities in patients presenting with clinical and biochemical features listed in Table 1.

**Table 1:** Clinical and biochemical features requiring IEF of serum transferrin.

**Non-immune hydrops foetalis (NIHF)**
**Inverted nipples, abnormal fat distribution**
**Connective tissue involvement (*cutis laxa*, inguinal hernias)**
**Unexplained multisystemic phenotype, including neurological manifestation**
**Non-progressive cerebellar ataxia**
**Severe epileptic encephalopathy**
**Elevated serum transaminases (especially with decreased antithrombin/protein C and S activity)**
**Liver steatosis/fibrosis/cirrhosis of unknown etiology**
**Recurrent pericardial effusion**
**Cardiomyopathy**
**Skeletal dysplasia (especially features of pseudodiastrophic dysplasia, Gillessen-Kaesbach and Nishimura skeletal dysplasia, Desbuquois dysplasia, brachytelephalangy)**
**Immunodeficiency**

Transferrin (Tf) is a plasma iron transport protein with two asparagine N-glycosylation sites (Asn432 and Asn630), and the dominated isoform in healthy individuals is tetrasialo-Tf, while asialo- and monosialo-Tf isoforms are usually not detectable. A type 1 pattern (CDG-I) is associated with an increased asialo- and disialo-Tf, and decreased tetrasialo-Tf, indicating an assembly or transfer defect of the dolichol-linked glycan (85). A type 2 pattern (CDG-II) is associated with increased asialo-, monosialo-, disialo-, and trisialo-Tf, indicating a processing defect after glycan transfer in the ER or during Golgi glycosylation (85). PGM1-CDG presents features of the both types of serum Tf IEF patterns (mixed type, CDG-I/CDG-II).

During CDG diagnostic process, it is important to exclude secondary causes of N-hypoglycosylation as well as Tf gene polymorphisms. Several Tf gene polymorphisms (i.e., transferrin B2 presenting with an elevated pentasialo-Tf solely or transferrin C2 resulting in increased trisialo-Tf solely) are known to result in a shifted IEF pattern, caused by pI differences of the polypeptide chain.

It is also known that untreated patients with classic galactosemia (galactose-1-phosphate uridyltransferase deficiency) and fructosemia (fructose 1-phosphate aldolase deficiency) have secondarily an abnormal serum Tf isoform profile that could resemble CDG-I. For example, Bogdańska et al. reported a study on 19 pediatric patients with primary liver disease and increased secondary asialo-Tf and monosialo-Tf isoforms; none of the patients had an elevated level of trisialo-Tf isoform (97). On the other hand, Jansen et al. published a study about secondary glycosylation defects in 961 adult patients qualified for LTx or with chronic liver disease. It showed that 247 patients (26%) had hyposialylation of serum Tf, while the majority of them (70%) had an increase in the trisialo-Tf isoform (98).

Normal serum Tf IEF profile does not exclude CDG—we should consider targeted next-generation sequencing (NGS) or whole-exome sequencing (WES) in case of a strong clinical and biochemical suspicion. PMM2-CDG due to promotor defect and several other CDG, like SLC35A1-CDG, SLC35A3-CDG, SEC23B-CDG, and PGM3-CDG, could show normal N-glycosylation profile. What is more, serum Tf IEF could be normal as well as abnormal in several others CDG, like ALG13-CDG, SLC35A2-CDG, RTF1-CDG, and SRD5A3-CDG (1–4, 85).

After the diagnosis of CDG-I based on serum Tf IEF, phosphomannomutase-2 (PMM2) and phosphomannose isomerase (PMI) activity should be measured in fibroblasts or leukocytes in the proper clinical context (1–4, 85). PMM2-CDG has the best-defined clinical phenotype and is by far the most frequent N-glycan assembly defect (99). PMI activity should be measured in case of clinical and biochemical presentation mainly expressed by the liver. If the clinical phenotype is not typical for PMM2-CDG and MPI-CDG, and in the case of normal PMM2 and PMI activity, plasma N-glycan analysis by MS (total plasma and/or intact transferrin glycoprofiling) could be done (1–4, 85). Nowadays, this is replaced by next-generation sequencing (NGS), including targeted NGS (panel of genes known to be involved in CDG) or whole exome sequencing (WES) (Figure 1). However, since NGS became more widely available, an improvement in diagnostics has been observed, with more patients and novel CDG subtypes being reported. These molecular analyses could take more time to result and be more expensive than laboratory CDG screening, although the high-throughput methods (like MS) are not routinely available (like in our center) and require both a qualified staff and comprehensive equipment.

After the diagnosis of CDG-II, IEF of serum apolipoprotein C-III (apoC-III) is recommended to perform (Figure 1) to distinguish between an exclusive N-glycosylation defect and a combined disorder of N- and O-glycosylation (1–4, 85). This analysis was described by Wopereis et al. in 2003 as a screening method for defects in the biosynthesis of the core 1 mucin-type O-glycans (100). However, some patients could also have an abnormal biosynthesis of core 1 O-glycans, including those with hemolytic uremic syndrome due to *Streptococcus pneumoniae*. Therefore, other laboratory methods have been developed parallel to serum Tf IEF, including serum apoC-III CZE.

Protein-linked glycan analysis should next be performed in attempt to identify the defective step, or targeted NGS, or WES (94).

Molecular analysis is necessary to confirm the final diagnosis of CDG and predict the possible genotype-phenotype correlation. However, the combination of MS with clinical exome sequencing (especially WES) is helpful to identify new CDG defects.

## Treatment

An early diagnosis of CDG is crucial for the timely implementation of appropriate therapies. However, causative treatment is available only for few CDG types in the form of specific monosaccharide supplementation therapy (i.e., galactose for PGM1-CDG, fucose for SLC35C1-CDG, Mn^2+^ for TMEM165-CDG, or mannose for MPI-CDG) (101–103). For the majority of patients, only symptomatic treatment can be offered. The natural history for most CDG types is unknown (also due to lack of long-term follow-up); however, cerebellar ataxia in PMM2-CDG is not progressive, and patients could even slowly improve with age (26, 27).

Several therapeutic strategies were developed for PMM2-CDG, including mannose supplementation, inhibition of MPI, pharmacological chaperones, proteostasis regulators (celastrol), acetazolamide, and antisense therapy (104). To date, no causative treatment for PMM2-CDG exists. However, acetazolamide was reported to be well tolerated and effective for cerebellar syndrome (105). In addition, Taday et al. recently published a study on long-term oral mannose supplementation in 20 patients with PMM2-CDG (106). The therapy was tolerated well, and biochemical improvement was noted in the majority of patients.

Symptomatic treatment in PMM2-CDG includes:

- nutritional support (including percutaneous endoscopic gastrostomy placement) in failure-to-thrive patients;
- regular albumin infusions, octreotide therapy, or a diet rich in mid-chain fatty acids (MCTs) in protein-losing enteropathy;
- levothyroxine in the presence of decreased free thyroxine;
- fresh frozen plasma infusions to prevent bleeding episodes;
- pleural-pericardial window formation in recurrent pericardial effusion (26, 36).

The administration of mannose in MPI-CDG improves the clinical and biochemical outcome (including serum transferrin isoforms); however, patients can still develop progressive liver fibrosis (107–110). Mannose therapy in MPI-CDG was also discontinued in a few patients due to side effects (40). One reported patient with MPI-CDG required liver transplantation due to chronic liver disease with the development of hepatopulmonary syndrome (39).

Two patients with CCDC115-CDG underwent LTx; one rejected the transplant and died while the other is doing well, showing biochemical improvement of liver function tests and transferrin glycosylation profile (111).

Galactose therapy in PGM1-CDG is safe and associated with a significant improvement of *N*-glycosylation and clinical parameters (liver function tests, coagulation, blood glucose) (38, 77). Four patients with PGM1-CDG underwent heart transplantation, and all died due to cardiac disease-related complications. Three other patients reported by Tegtmeyer et al. were listed for heart transplantation (38).

Heart transplantation could be considered as a treatment option in other patients with cardiac involvement. It was performed in three patients with defects in dolichol synthesis (DOLK-CDG, DK1-CDG), despite supportive heart failure therapy (ACE inhibitors, β-blockers, and diuretics); one of them died unexpectedly 2 years after transplantation at the age of 16.5 years (51).

Besides PGM1-CDG, galactose supplementation showed promising results in SLC35A2-CDG, SLC39A8-CDG, and TMEM165-CDG (112–114).

Fucose supplementation in SLC35C1-CDG was reported to decrease infection rates, normalize neutrophil counts, and improve psychomotor development. However, it should be monitored carefully due to the risk of autoimmune and hemolytic reactions (115–117).

Uridine supplementation in CAD-CDG patients was reported to improve the clinical manifestation, including seizure cessation, cognitive and motor development, and normalization of biochemical parameters (118).

Hematopoietic stem cell transplantation was successfully applied to treat CDG with immunodeficiency in PGM3-CDG children (57).

## Author Contributions

PL and AT-S: project administration and writing—review and editing. AT-S: supervision. PL: writing—original draft. Both authors contributed to the article and approved the submitted version.

## Conflict of Interest

The authors declare that the research was conducted in the absence of any commercial or financial relationships that could be construed as a potential conflict of interest.

## Publisher's Note

All claims expressed in this article are solely those of the authors and do not necessarily represent those of their affiliated organizations, or those of the publisher, the editors and the reviewers. Any product that may be evaluated in this article, or claim that may be made by its manufacturer, is not guaranteed or endorsed by the publisher.
